# Suicide-related stigma and its relationship with help-seeking, mental health, suicidality and grief: scoping review

**DOI:** 10.1192/bjo.2024.857

**Published:** 2025-03-21

**Authors:** Jessica M. Wyllie, Kathryn A. Robb, David Sandford, Marianne E. Etherson, Nadia Belkadi, Rory C. O’Connor

**Affiliations:** 1 Suicidal Behaviour Research Laboratory, School of Health and Wellbeing, Clarice Pears Building, University of Glasgow, Glasgow, UK; 2 School of Health and Wellbeing, Clarice Pears Building, University of Glasgow, Glasgow, UK; 3 School of Health, Social Work and Sport, Brook Building, University of Central Lancashire, Preston, UK

**Keywords:** Suicide, stigma and discrimination, self-harm, prevention, scoping review

## Abstract

**Background:**

Suicide-related stigma (i.e. negative attitudes towards people with suicidal thoughts and/or behaviours as well as those bereaved by suicide) is a potential risk factor for suicide and mental health problems. To date, there has been no scoping review investigating the association between suicide-related stigma and mental health, help-seeking, suicide and grief across several groups affected by suicide.

**Aims:**

To determine the nature of the relationship between suicide-related stigma and mental health, help-seeking, grief (as a result of suicide bereavement) and suicide risk.

**Method:**

This review was registered with PROSPERO (CRD42022327093). Five databases (Web of Science, APA PsycInfo, Embase, ASSIA and PubMed) were searched, with the final update in May 2024. Studies were included if they were published in English between 2000 and 2024 and assessed both suicide-related stigma AND one of the following: suicide, suicidal thoughts or suicidal behaviours, help-seeking, grief or other mental health variables. Following screening of 14 994 studies, 100 eligible studies were identified. Following data charting, cross-checking was conducted to ensure no relevant findings were missed.

**Results:**

Findings across the studies were mixed. However, most commonly, suicide-related stigma was associated with higher levels of suicide risk, poor mental health, lowered help-seeking and grief-related difficulties. A model of suicide-related stigma has been developed to display the directionality of these associations.

**Conclusions:**

This review emphasises the importance of reducing the stigma associated with suicide and suicidal behaviour to improve outcomes for individuals affected by suicide. It also identifies gaps in our knowledge as well as providing suggestions for future research.

Suicide, the act of a person taking their own life, is a major public health concern worldwide, with around 720 000 people dying by suicide each year and many more attempting suicide.^1^ There are many risk factors associated with suicide;^2–4^ however, this review aims to focus on one such potential risk factor, suicide-related stigma, which historically has not received the attention it deserves. The American Psychological Association (APA) defines stigma as ‘the negative social attitude attached to a characteristic of an individual that may be regarded as a mental, physical, or social deficiency’.^5^ This scoping review focuses specifically on suicide-related stigma, which can be described as negative attitudes and behaviours towards those who have experienced suicidal ideation or attempted suicide or have been bereaved by suicide. These negative attitudes and behaviours can manifest as unconscious biases, stereotyping, prejudice and discrimination.^6,7^ Furthermore, suicide-related stigma can take the form of self-stigma (internalised stigma that people hold about themselves^6,8,9^), public stigma (stigma held by members of society about suicide^8^) or anticipated and/or perceived stigma (the fear of being discriminated against owing to stigma^8^).

## Theoretical models

To our knowledge, there are no specific suicide-related stigma models. There are, however, several relevant models and theories which can aid our understanding of mental health stigma’ these include the modified labelling theory (MLT),^10^ the social cognitive model,^11,12^ the stereotype content model,^13,14^ the dual process model^15^ and attribution theory.^16^ Those most relevant here are the MLT,^10^ which explains public stigma, and the social cognitive model,^11,12^ which focuses on the development of self-stigma as a result of public stigma. The MLT^10^ proposes that when members of society view those with mental illnesses negatively, this leads to the devaluation of the individual with a mental illness. For example, when people with mental illnesses are labelled ‘abnormal’ or ‘crazy’, they can be discriminated against and excluded as a result. The theory also posits that the perception or anticipation of being negatively labelled or stereotyped affects the way individuals view themselves, leading to secrecy and social withdrawal or, in some cases, to a desire to educate others about mental illness. This is further supported by a meta-analysis by Livingston and Boyd,^17^ who found that higher perceptions of stigma among people with a mental illness were associated with reduced social support. The social cognitive model^11,12^ builds on the MLT^10^ and explains how stigma is composed of stereotypes, prejudices and discrimination directed at those with a mental illness. The model focuses on how these stereotypes can be internalised, leading to harmful beliefs about oneself. The social cognitive model^11,12^ explores how individuals can engage in behaviours such as label avoidance to avoid experiencing public stigma (i.e. individuals avoid seeking help to avoid being labelled as having a mental illness and becoming part of a stigmatised group). It is also worth briefly mentioning attribution theory,^16^ which posits that those who are perceived as responsible for their stigmatised identity are more likely to be subject to higher levels of stigma. This relates to those who have a suicidal history, because often people refer to suicide as a choice.^18,19^ As a result, these individuals are often viewed as being personally responsible and thus more likely to be stigmatised to a greater degree compared with individuals who are not viewed as personally responsible for their stigmatised identity.

More general suicide models are also relevant here, with the interpersonal theory of suicide^20^ being of particular interest, as some of its components (thwarted belongingness, perceived burdensomeness) may have their roots in stigma. This theory argues that suicidal behaviour is more likely to occur when an individual has both the desire to die and the capability to carry out the act. It posits that this desire often emerges from feelings of loneliness, a lack of belongingness and perceived burdensomeness. In the present context, it is reasonable to predict that these factors may emerge as a result of suicide-related stigma, as stigma ‘can lead unfairly to discrimination against and exclusion of the individual’.^5^ The role of suicide-related stigma also fits with another theoretical model, the integrated motivational–volitional (IMV) model of suicide.^21,22^ The IMV model comprises three phases, of which the second (motivational) phase is most relevant here. This phase focuses on an individual’s sense of defeat, humiliation and entrapment, which are described as predictors of suicidal ideation. Such feelings of defeat and entrapment can also be experienced through social rejection or loss.^23^ Furthermore, the IMV model^22^ suggests that so-called motivational moderators can increase or decrease the likelihood that entrapment acts as a precursor to suicidal ideation. Motivational moderators include feelings of belongingness and connectedness, among others, and can be protective; however, their absence can mean that an individual is at greater risk of suicide.^22^ Thus, we posit that the exclusion of an individual as a result of the stigmatisation associated with their experiences of suicidal thoughts or behaviours could cause them to feel lonely and isolated, thereby increasing their risk of suicide.^24,25^

## Pre-existing research

The number of individuals affected by suicide is staggering, with research showing that over the course of the lifespan, one person in five experiences suicidal thoughts and one in 15 attempts suicide.^26^ Furthermore, research has shown that the impact of an individual suicide can be widespread, with some estimates suggesting that as many as 135 individuals are potentially affected by each suicide death.^27^ The research on risk factors for suicide is vast, with thousands of papers highlighting the influence of poor mental health, lowered help-seeking behaviours and grief as risk factors for suicide. However, there is arguably a lack of understanding of the nature of the relationship between suicide-related stigma and these risk factors. The existing literature in this area has identified suicide-related stigma as an important factor in suicide; however, few review articles have explored this relationship and its direction in conjunction with other suicide risk factors such as lowered help-seeking, grief and mental health. It is important to clarify the extent to which suicidal thoughts and behaviours lead to suicide-related stigma and vice versa. Two systematic reviews in this area have focused on the relationship between suicide-related stigma and those bereaved by suicide.^9,28^ They found that individuals bereaved by suicide reported feeling shamed, blamed and judged for the loss of their loved one to suicide, and that these feelings often had negative consequences. Those who felt stigmatised as a result of being bereaved by suicide were also more likely to keep the suicide a secret, withdraw from social situations, and suffer from depression and complicated grief, as well as being at greater risk of self-harm and suicide.^9,28^

## Aims

Given the broad nature of our research questions, a scoping review was most appropriate. Although the aforementioned systematic reviews are relatively recent, the literature on suicide-related stigma and those bereaved by suicide has grown considerably in recent years. In addition, this scoping review addresses suicide-related stigma among those who have attempted suicide or experienced suicidal ideation, as well as those who have experience of suicide in their professional and/or personal lives (i.e. in professional settings or through having family or friends who did not die by suicide) and those with no experience of suicide (general population). To our knowledge, no previous review has explored the association between suicide-related stigma and the experiences of individuals who have attempted suicide or experienced suicidal ideation; the existing reviews focus solely on those bereaved by suicide. Furthermore, this review includes studies where suicide-related stigma is the outcome variable and those where it is the exposure variable. This allows for a thorough understanding of the direction of the associations between suicide-related stigma and suicide, help-seeking, grief and mental health. In short, this scoping review addresses a gap in the research literature, as it aims to explore the role of stigma associated with suicidal behaviour across the three groups affected by suicide (i.e. suicidal ideation, suicide attempt and bereavement groups) as well as general population samples, and the relationships of stigma with help-seeking, grief and mental health. Specifically, this review investigates the following three research questions.

- Is there evidence that suicide-related stigma is associated with suicide risk (suicide, suicidal thoughts and suicidal behaviours), help-seeking, grief and mental health?
- Is there evidence that people with different exposures to suicide report different levels of suicide-related stigma?
- What are people’s experiences of suicide-related stigma in the context of suicide risk, help-seeking, grief and mental health?

## Methods

The PRISMA-ScR (Preferred Reporting Items for Systematic Reviews and Meta-Analyses extension for Scoping Reviews)^29^ guidelines were followed for this scoping review (Supplementary Material 1, available at https://doi.org/10.1192/bjo.2024.857). The protocol has been published and can be accessed on Prospero (CRD42022327093). This review was originally registered as a systematic review; however, following feedback from reviewers, we changed it to a scoping review.

### Search strategy

A keyword search was conducted using the following databases: Web of Science Core Collection, APA PsycInfo – EBSCOHost, Embase (Ovid), ASSIA: Applied Social Sciences Index & Appendices and PubMed. The final search was conducted on 2 May 2024. Searches were set to include empirical articles published in English between the years 2000 and 2024. The decision to start the search at the year 2000 was informed by the World Health Organization’s ‘*Preventing Suicide: A Global Imperative*’ report^,30^ which states that several national suicide prevention strategies have been developed since the year 2000. Further, the World Health Organization reported that in the past 15 years, the delivery of training packages on suicide prevention has become widespread, and there has been a substantial increase in the number of self-help groups for those who have attempted suicide or those bereaved by suicide. As a result, the stigma associated with suicide and suicidal behaviour is likely to have changed in the past 20+ years, hence our decision to focus on studies published from 2000 onwards. Keyword searches included but were not limited to the terms ‘suicide’, ‘stigma’ and ‘attitudes’; as an example, the search strategy used for the APA PsycInfo database is included in Supplementary Material 2. After database searches had been conducted, de-duplication was performed before the screening stages. The references of the studies included were manually searched to identify studies missed by the database search.

### Eligibility criteria

The inclusion criteria were:

- empirical studies;
- studies available in English;
- studies published in and after the year 2000;
- all age groups;
- qualitative and quantitative studies;
- studies assessing both suicide-related stigma AND one of the following: suicide, suicidal thoughts or suicidal behaviours, help-seeking, grief or other mental health variables (such as depression).

The exclusion criteria were:

- studies published before the year 2000;
- studies focusing on non-suicidal self-harm only;
- systematic reviews, narrative reviews, meta-analyses or book chapters;
- abstract-only publications.

### Data charting

J.M.W. completed title and abstract screening and full-text screening. Upon completion of each screening stage by J.M.W., a random sample of 20% of all studies was cross-checked by reviewers (D.S. and M.E.E.) against the eligibility criteria, and any discrepancies were discussed. Data extraction began on 15 August 2022 and was conducted by J.M.W.; 100% of the results were then cross-checked by all other reviewers (D.S., M.E.E. and N.B.) to ensure all relevant data had been extracted. A pre-existing data extraction sheet was used by all reviewers at this stage (Supplementary Material 3).

### Data synthesis

As studies included within this review used a wide range of methods, a narrative synthesis was the most appropriate way to analyse the quantitative findings. Thematic synthesis^31^ informed our analysis of the qualitative papers. This involved coding the findings of the included papers to create descriptive themes, followed by the development of analytical themes to determine common themes across the papers. Given the study heterogeneity, a meta-analysis was not possible. Quantitative papers were assessed; then, qualitative papers were assessed separately. Next, links were determined between the findings; see the Discussion section for details of these links. Included studies were organised by the research question that they were answering and further by suicide risk, mental-health, help-seeking and grief. For mixed-methods studies, quantitative results are presented with those of the quantitative studies, and qualitative results are presented with those of the qualitative studies. Results were further organised by study population (i.e. general population, those with experience of suicide in their professional and/or personal lives, those with a history of suicidal ideation, suicide attempt survivors, those bereaved by suicide). Studies under the heading ‘General population’ were those in which participants in the sample did not report experience of suicide (personally and/or professionally), or in which less than 40% of the sample reported experience of suicide in their personal or professional lives.

## Results

**Fig. 1:**
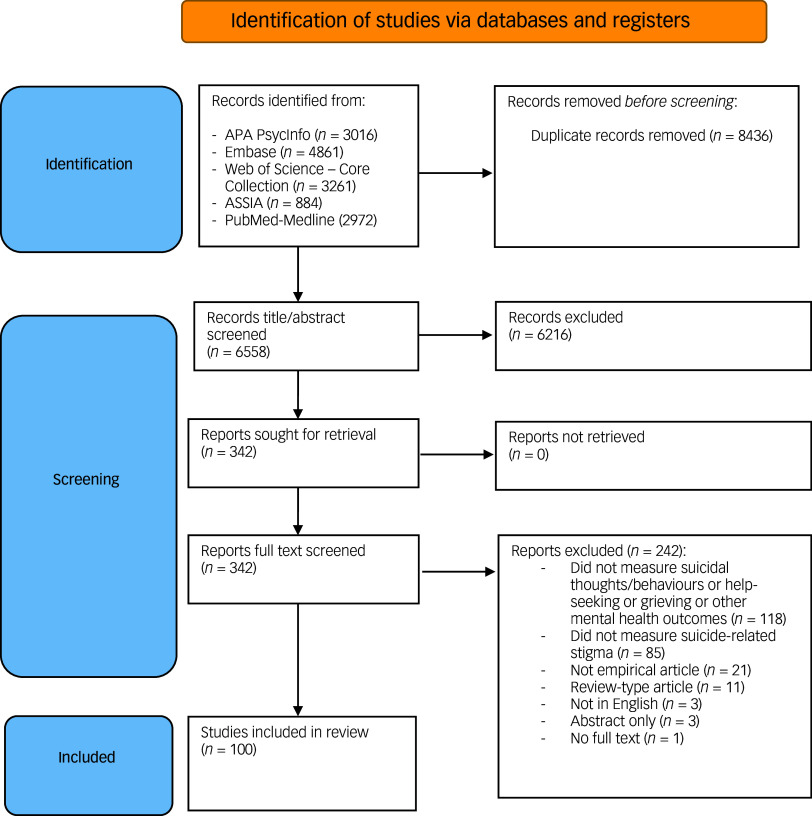
Flowchart of selection process.

The searches yielded 14 994 studies; after de-duplication, 6558 unique records remained, and these were screened (Fig. 1). After screening titles and abstracts, 342 full-text studies remained and were further screened for eligibility based on full-text review. In total, 100 studies met the inclusion criteria and were included in this scoping review.

### Study characteristics

Of the 100 included studies, 52 were quantitative, 45 were qualitative and three employed mixed-methods designs. The included studies were conducted in a range of countries; however, they were most commonly conducted in the USA (*n* = 22). Table 1 provides author information and study characteristics for each included paper. More than 70% (*n* = 71) of studies comprised predominantly females. The type and measure of suicide-related stigma investigated varied across studies; the most commonly used measure in the quantitative studies (50%, *n* = 26) was the Stigma of Suicide Scale (SOSS)^32^ in both its long form and a short form (SOSS-SF).

Table 1 provides author information and relevant characteristics of each study included in the synthesis.

**Table 1:** Author information and characteristics

Study number	Author	Design	Sample size (country)	Measure
**1**	Al Shannaq and Aldalaykeh (2021)^50^	Quantitative	707 (Jordan)	SOSS-SF
**2**	An Fhaili et al (2016)^114^	Qualitative	15 (Ireland)	Focus group
**3**	An and Lee (2019)^73^	Quantitative	1599 (Korea)	AM
**4**	Arafat et al (2022)^77^	Quantitative	529 (Bangladesh)	SOSS
**5**	Asare-Doku et al (2017)^110^	Qualitative	10 (Ghana)	Interview
**6**	Azizpour et al (2018)^97^	Qualitative	7 (Iran)	Interview
**7**	Azorina et al (2019)^115^	Qualitative	499 (UK)	Online survey
**8**	Batterham et al (2013)^32^	Quantitative	1286 (Australia)	SOSS-SF
**9**	Batterham et al (2019)^67^	Quantitative	1697 (Australia)	SOSS-SF
**10**	Binnix et al (2018)^95^	Qualitative	20 (–)	Interview
**11**	Blanchard and Farber (2020)^96^	Qualitative	66 (USA)	Short essay
**12**	Denmark et al (2012)^129^	Qualitative	558 (USA)	Online survey
**13**	Williams et al (2018)^54^	Quantitative	499 (–)	SOSS-SF/SOQ
**14**	Calear et al (2014)^46^	Quantitative	1274 (Australia)	SOSS-SF
**15**	Cerel et al (2006)^69^	MM	679 (USA)	–
**16**	Chan et al (2014)^55^	Quantitative	895 (Australia)	SOSS
**17**	Doria et al (2021)^89^	Qualitative	73 (USA)	Focus group
**18**	Feigelman et al (2021)^52^	Quantitative	195 (USA/Canada)	STOSASS
**19**	Fielden (2003)^116^	Qualitative	6 (New Zealand)	Interview
**20**	Freedenthal and Stiffman (2007)^130^	Qualitative	101 (–)	Interview
**21**	Frey et al (2016)^85^	Quantitative	156 (USA)	AM
**22**	Frey et al (2017)^98^	Qualitative	40 (USA)	Interview
**23**	Fulginiti and Frey (2018)^33^	Quantitative	26 (USA)	STOSA
**24**	Geležėlytė et al (2020)^48^	MM	82 (Lithuania)	GEQ
**25**	Guerrero-Diaz et al (2021)^56^	Quantitative	135 (Spain)	CCCS-18
**26**	Han et al (2017)^70^	Quantitative	224 (China)	SOSS-SF
**27**	Harwood et al (2002)^79^	Quantitative	85 (UK)	GEQ
**28**	Hom et al (2019)^34^	Quantitative	818 (USA)	SOSS-SF
**29**	Kearns et al (2015)^42^	Quantitative	493 (Ireland)	STOSA
**30**	Kheibari et al (2021)^57^	Quantitative	504 (USA)	SOSS-SF
**31**	Kheibari et al (2022)^84^	Quantitative	312 (USA)	SOSS/SOSS-SF
**32**	Kolves et al (2019)^80^	Quantitative	205 (Australia)	GEQ
**33**	Kolves et al (2020)^78^	Quantitative	205 (Australia)	GEQ
**34**	Ludwig et al (2022)^58^	Quantitative	2002 (Germany)	SOSS-SF
**35**	Maple et al (2010)^117^	Qualitative	22 (Australia)	Interview
**36**	Maple et al (2020)^99^	Qualitative	31 (Australia)	Interview
**37**	Mayer et al (2020)^35^	Quantitative	159 (USA)	AM
**38**	Mugisha et al (2011)^90^	Qualitative	– (Uganda)	Interview
**39**	Murphy et al (2019)^59^	Quantitative	396 (Canada/Australia)	ATTS/SOSS-SF
**40**	Oexle et al (2020)^38^	Quantitative	195 (91% USA)	STOSASS
**41**	Oexle (2019)^100^	Qualitative	13 (Germany)	Interview
**42**	Ohayi (2019)^118^	Qualitative	27 (Nigeria)	Interview
**43**	Osafo et al (2015)^108^	Qualitative	10 (–)	Interview
**44**	Park et al (2015)^76^	Quantitative	1522 (Korea)	PDD
**45**	Peters et al (2016)^119^	Qualitative	10 (–)	Interview
**46**	Pitman et al (2016)^81^	Quantitative	3432 (UK)	GEQ
**47**	Pitman et al (2018)^120^	Qualitative	27 (UK)	Interview
**48**	Prawira and Sukmaningrum (2020)^51^	Quantitative	284 (Jakarta)	SOSS
**49**	Richards et al (2019)^131^	Qualitative	26 (USA)	Interview
**50**	Rimkeviciene et al (2015)^101^	Qualitative	15 (Australia)	Interview
**51**	Rimkeviciene et al (2021)^36^	Quantitative	3947 (Australia)	PSSQ
**52**	Ross et al (2021)^121^	Qualitative	15 (Australia)	Focus group
**53**	Scocco et al (2012)^74^	Quantitative	527 (Italy)	STOSA/STOSASS
**54**	Scocco et al (2019)^53^	Quantitative	240 (Italy)	STOSSS
**55**	Scocco et al (2017)^83^	Quantitative	155 (Italy)	STOSSS
**56**	Scocco et al (2016)^75^	Quantitative	67 (Italy)	STOSA
**57**	Sheehan et al (2018)^102^	Qualitative	62 (USA)	Focus group
**58**	Sheehan et al (2020)^72^	Quantitative	292 (USA)	SSSAS
**59**	Sheehan et al (2017)^6^	Qualitative	62 (USA)	Focus group
**60**	Tzeng et al (2010)^122^	Qualitative	15 (Taiwan)	Interview
**61**	Vatan et al (2010)^71^	Quantitative	749 (USA/Turkey)	AM
**62**	Wu et al (2021)^63^	Quantitative	412 (China)	STOSA/STOSASS/SOSS-SF
**63**	Yakunina et al (2010)^43^	Quantitative	321 (USA)	SSS/SOQ
**64**	Wahab et al (2021)^86^	Quantitative	290 (Malaysia)	ATTS
**65**	Sheehan et al (2019)^93^	Qualitative	40 (USA)	Interview/Focus group
**66**	Eskin et al (2011)^64^	Quantitative	646 (Austria/Turkey)	AM
**67**	Fong et al (2022)^65^	Quantitative	1946 (Hong Kong)	SOSS-SF
**68**	Gaffney and Bergmans (2022)^103^	Qualitative	8 (Canada)	Interview
**69**	Ruzhenkov et al (2015)^37^	Quantitative	125 (Russia)	AM
**70**	Chapple et al (2015)^111^	Qualitative	80 (UK)	Interview
**71**	Roskar et al (2022)^45^	Quantitative	83 (Slovenia)	PSSQ
**72**	Ali and Rehna (2022)^123^	Qualitative	6 (Pakistan)	Interview
**73**	Sever and Ozdemir (2022)^124^	Qualitative	7 (Turkey)	Interview
**74**	Oexle et al (2022)^112^	Qualitative	22 (Germany)	Interview
**75**	Heard et al (2022)^125^	Qualitative	23 (Australia)	Focus group
**76**	Mayer et al (2023)^104^	Qualitative	22 (Germany)	Interview
**77**	Zou et al (2022)^126^	Qualitative	30 (China)	Interview
**78**	Freeman et al (2022)^105^	Qualitative	35 (Australia)	Focus group
**79**	Islam et al (2024)^60^	Quantitative	162 (Bangladesh)	SOSS
**80**	Ünsal and İnan (2024)^109^	Qualitative	16 (Turkey)	Interview
**81**	Burke et al (2023)^44^	Quantitative	302 (Australia)	SOSS-SF
**82**	Calear et al (2024)^61^	Quantitative	1019 (Australia)	SOSS-SF
**83**	Sharwood et al (2023)^68^	Quantitative	5426 (Australia)	SOSS
**84**	Fong and Yip (2023)^49^	Quantitative	2022 (Hong Kong)	SOSS
**85**	Goulah-Pabst (2023)^94^	Qualitative	14 (USA)	Interview
**86**	Gupta et al (2023)^88^	Quantitative	319 (Nepal)	SOSS-SF
**87**	Jahan et al (2023)^66^	Quantitative	616 (Bangladesh)	SOSS-SF
**88**	Prawira et al (2023)^41^	Quantitative	484 (Indonesia)	SOSS-SF
**89**	Collado et al (2023)^62^	Quantitative	466 (Spain)	SOSS-SF
**90**	Richardson et al (2023)^113^	Qualitative	13 (Ireland)	Interview
**91**	Anderson et al (2024)^106^	Qualitative	16 (UK)	Interview
**92**	Chen et al (2023)^127^	Qualitative	5 (China)	Interview
**93**	Lyu and Li (2023)^40^	Quantitative	944 (China)	SOSS-SF
**94**	Maclean et al (2023)^47^	Quantitative	140 (Australia)	PSSQ
**95**	Osafo et al (2012)^91^	Qualitative	17 (Ghana)	Interview
**96**	Osafo et al (2019)^92^	Qualitative	10 (Ghana)	Interview
**97**	Williams et al (2018)^54^	Qualitative	25 (USA)	Interview
**98**	Domino et al (2002)^39^	MM	104 (China/USA)	SOQ
**99**	Groh et al (2018)^128^	Qualitative	10 (Guyana)	Focus group
**100**	Skogstad et al (2005)^132^	Qualitative	52 (New Zealand)	Interview

MM, mixed methods; GEQ, Grief Experience Questionnaire; AM, Adapted Measure; SOSS/SOSS-SF, Stigma of Suicide Scale/Short-form; SOQ, Suicide Opinion Questionnaire; STOSA, Stigma of Suicide Attempt; ATTS, Attitudes towards Suicide Scale; PDD, Perceived Devaluation and Discrimination Scale; PSSQ, Personal Suicide Stigma Scale; STOSSS, Stigma of Suicide Survivor Scale; SSSAS, Self-Stigma of Suicide Attempt Scale; SSS, Stigma of Suicide Scale; CCCS-18, Spanish version of Attitudinal Beliefs Questionnaire about Suicidal Behavior; –, not reported.

The qualitative and quantitative findings are presented under the relevant research questions in the sections below. In addition, a synthesis of key themes is summarised in Table 2.

**Table 2:** Key themes identified across qualitative studies and the corresponding findings from quantitative studies

	Suicide group		
Key themes	Exposed to suicide	Suicide ideation/ attempt	Suicide survivors
Negative labelling	**●**	**●**	**●**
Fear	**●**	**●**	
Shame	**●**	**●**	● ▪
Contamination	**●**		
Secrecy		● ▪	● ▪
Rejection/Isolation		● ▪	● ▪
Judgement		**●**	**●**
Blame/Guilt		● ▪	● ▪
Sinful/Disapproving		●	●
Discrimination		● ▪	

●= Qualitative ▪ = Quantitative.

### Overview of the findings

**Fig. 2:**
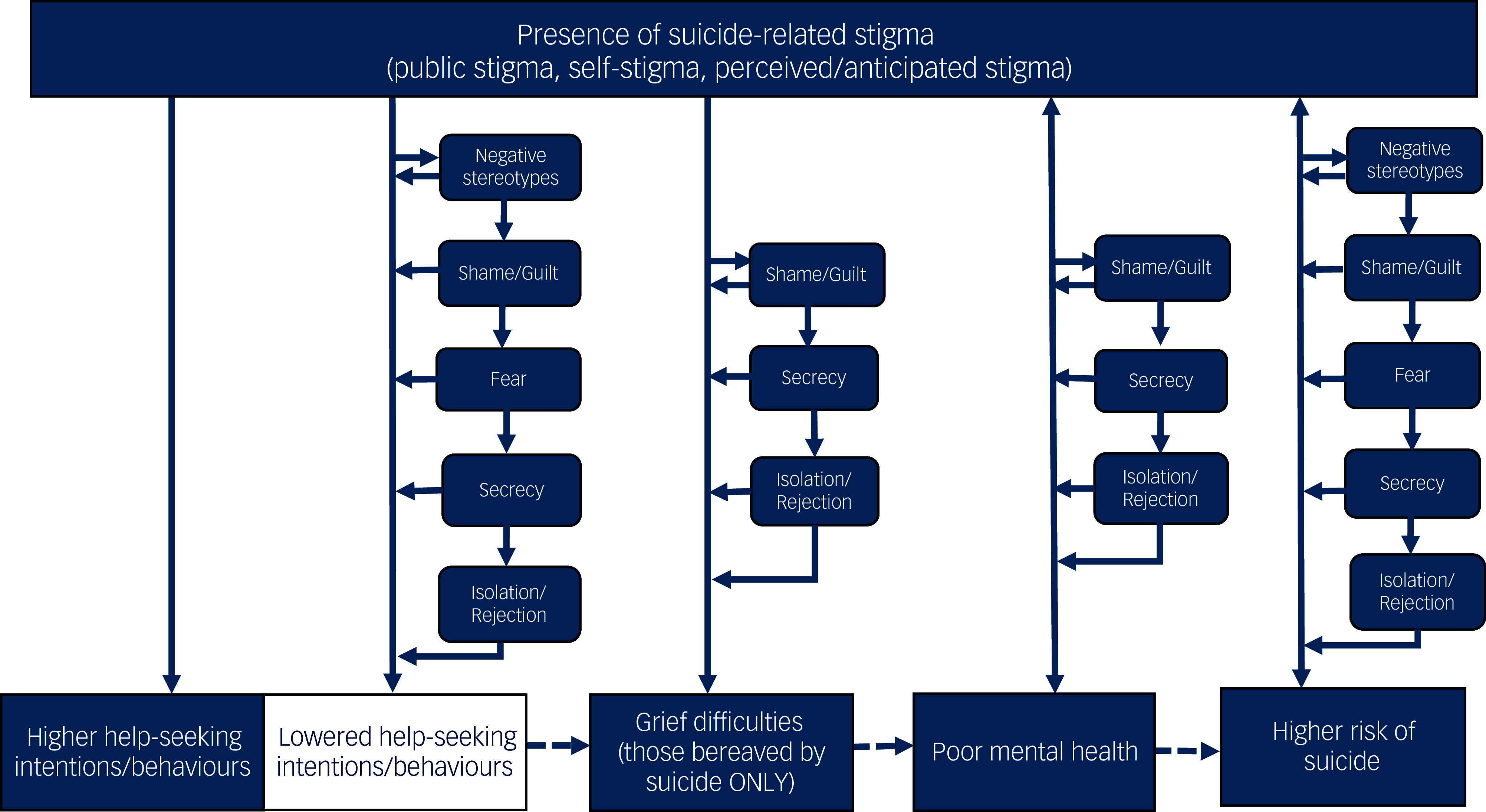
Presence of suicide-related stigma (public stigma, self-stigma, and perceived and/or anticipated stigma).

A pathway model of suicide-related stigma is outlined in Fig. 2; this helps to summarise the different pathways described in the findings of this scoping review. The figure displays the proposed directions of the relationships between suicide-related stigma and help-seeking behaviours, grief, mental health and suicide risk using arrows. The findings suggest that both higher and lower levels of help-seeking, together with grief, have unidirectional relationships, whereas poor mental health and higher levels of suicide risk appear to have a bi-directional relationship with suicide-related stigma. In some studies, suicide-related stigma was associated with poorer mental health and suicide risk, but poor mental health and experience of suicide also predicted levels of suicide-related stigma. Within the qualitative studies, several factors, including negative stereotypes, shame and/or guilt, fear, secrecy, and isolation and/or rejection were reported as reasons that suicide-related stigma led to lowered help-seeking intentions or behaviours, grief difficulties (among those bereaved by suicide only), poor mental health and higher suicide risk. Furthermore, the four variables displayed at the bottom of the model in the figure (help-seeking, grief, mental health and suicide) all have dotted arrows connecting them; this is because, although not evidenced within this review, these variables are known to negatively affect one another. For example, lowered help-seeking has been shown to lead to grief related difficulties, which have been shown to lead to poor mental health, and poor mental health is a risk factor for suicide.

### Is there evidence that suicide-related stigma is associated with suicide risk (suicide, suicidal thoughts and suicidal behaviours), help-seeking, grief and mental health?

#### Suicide risk (suicide, suicidal thoughts and suicidal behaviours)

##### History of suicide attempt(s)

Five studies^33–37^ exploring suicide-related stigma as the exposure variable reported high levels of public suicide-related stigma and self-stigma among suicide attempt survivors. Of a sample of US suicide attempt survivors, 46% believed that their family members held stigmatising attitudes towards suicide attempt survivors.^33^ Twenty-nine per cent of participants in this sample had never told any of their family members about their suicide attempt, and, unsurprisingly, more perceived stigma about a suicide attempt was associated with greater concealment of a suicide attempt.^33^ Similar results were found in Russia,^37^ where participants reported feelings of rejection and dislike from family members owing to their suicide attempt. This study also found that many felt guilty and isolated as a result of their suicide attempt. Among a sample of suicide attempt survivors in Australia, individuals personally stigmatised their own suicidality.^36^ Although Hom et al^34^ did not find a significant relationship between suicide attempt history and suicide-related stigma, they did find public suicide-related stigma to be associated with an increased likelihood of future suicide attempts among US firefighters. Furthermore, anticipated stigma among suicide attempt survivors in the USA was associated with elevated risk of suicidal thoughts and behaviours even after controlling for time since last attempt and number of previous suicide attempts.^35^

##### Those bereaved by suicide

There was only one study which investigated suicide-related stigma as the exposure variable among those who had been bereaved by suicide. Among a mostly female sample of 195 individuals bereaved by suicide in the US (*n* = 180 females), higher levels of perceived stigma towards those bereaved by suicide were associated with increased suicidality and decreased personal growth.^38^

##### General population

In a sample of 104 Chinese and American women, viewing suicide as morally bad was associated with a greater risk of suicide.^39^ Moreover, in another sample of 944 Chinese students, public suicide-related stigma was found to inhibit suicide attempts but not suicidal ideation.^40^ Conversely, among a sample of Chinese Indonesians,^41^ public suicide-related stigma, in the form of viewing suicide as sinful, was associated with a decrease in an individual’s chances of experiencing self-harm or suicidal ideation.

#### Help-seeking

##### Experience of suicide in professional and/or personal life

Two studies conducted among students^42,43^ and one among parents with children who experienced suicidal thoughts^44^ found suicide-related stigma to be a significant predictor of factors related to help-seeking. The direction of the relationship varied as a function of the source of help. In one study conducted with suicidologists,^45^ no association was found. A study^42^ conducted with students in Ireland found that stigma towards suicide attempts was the strongest predictor of help-seeking stigma, whereby an increase in stigma towards suicide attempts was associated with an increase in help-seeking stigma. Another study^43^ of university students, conducted in the USA found that more public stigma towards suicide significantly increased help-seeking intentions from professional sources. However, the opposite relationship was found with regard to non-professional sources, in that more public suicide-related stigma was associated with a decrease in intentions to seek help from these sources.^43^ The results of these studies^42,43^ suggest that suicide-related stigma decreases the intention of seeking help from friends and family but not necessarily from professional sources. Research conducted in Australia^44^ found that parents who reported higher levels of public stigma towards suicide had more negative attitudes towards help-seeking for their child and lowered intentions to seek help for their child. All three studies which found suicide-related stigma to be associated with a decrease in help-seeking considered help-seeking among young adults^42,43^ or parents’ help-seeking behaviours on behalf of their children aged between 12 and 18 years old.^44^ By contrast, Roskar et al,^45^ who found no association between suicide-related stigma and help-seeking, conducted their research in Slovenia among suicidologists with an average age of 52 years. The studies also varied with respect to the type of suicide-related stigma measured; three studies^43–45^ measured public and perceived stigma towards those who die by suicide, whereas Kearns et al^42^ measured stigma towards suicide attempts.

##### Those with experience of suicidal ideation and/or behaviours

A study conducted with those who had past experiences of suicidal ideation in Australia found that increased public stigma towards people who die by suicide significantly predicted negative attitudes toward help-seeking and reduced intentions of seeking help from a mental health professional.^46^ Further research in Australia^47^ conducted among a sample of 140 individuals found that self-stigmatisation of suicidal behaviour was associated with decreased help-seeking intentions.

##### Those bereaved by suicide

In a single study of 81 individuals bereaved by suicide in Lithuania,^48^ those who sought help tended to have higher levels of perceived stigmatisation and guilt about the death of a loved one as a result of suicide. These individuals also had more positive attitudes toward seeking help.

##### General population

Three studies^49–51^ conducted among those with no history of suicide or exposure to suicide reported conflicting results. Al Shannaq and Aldalaykeh^50^ focused on 707 Arab youths and found a negative association between attitudes towards psychological help-seeking and public stigma towards suicide; however, when multivariable regression analyses were conducted, public stigma towards suicide was not a significant predictor of help-seeking. Among 2022 students from Hong Kong, higher levels of public stigma towards suicide were associated with lowered help-seeking behaviours.^49^ By contrast, research with 284 university students in Jakarta^51^ found that higher levels of public stigma towards those who die by suicide were associated with increased intentions to seek help, suggesting that suicide-related stigma was a protective factor within this sample.^51^

#### Grief

##### Those bereaved by suicide

Two studies from the USA found that perceived suicide-related stigma was associated with significantly greater grief-related difficulties^38,52^ and an increased likelihood of the suicide being kept a secret.^38^

#### Mental health

##### History of suicidal ideation and/or attempt(s)

Two studies^36,37^ with a combined total of 4072 participants reported that higher levels of personal stigmatisation of one’s own suicidality were associated with higher levels of psychological distress and/or psychotic symptoms.

##### Those bereaved by suicide

Among 240 people bereaved by suicide in Italy, levels of depression were positively associated with levels of perceived stigma towards those bereaved by suicide.^53^ Perceived stigma towards those bereaved by suicide was also found to be positively associated with feelings of shame and guilt and negatively associated with well-being among those bereaved by suicide in Lithuania.^48^

##### General population

One study, conducted with a large sample of 2022 young adults in Hong Kong, found that viewing suicide as selfish was significantly associated with higher levels of psychological distress.^49^

### Is there evidence that people with different exposures to suicide report different levels of suicide-related stigma?

#### Suicide risk (suicide, suicidal thoughts and suicidal behaviours)

##### Experience of suicide in professional and/or personal life

Across a range of populations (students, pharmacists, the general population and religious leaders), eight studies^54–61^ found suicide-related stigma to be lower in those with experience of suicide in their professional and/or personal lives. Conversely, among a sample of 466 Spanish individuals, those who had been exposed to a loss or a suicide attempt reported greater public stigma towards those who die by suicide compared with those who had no experiences of suicide.^62^ Further, the study by Murphy et al^59^ highlighted cultural differences, as more Australian than Canadian pharmacists endorsed the measure that those who die by suicide are cowardly, irresponsible and disconnected. A final study^63^ conducted among Chinese students found no association between stigma towards suicide attempts or those bereaved by suicide and levels of exposure to suicide.

##### History of suicidal ideation

Six studies found suicidal ideation to be associated with lower levels of public stigma towards those who die by suicide,^32,54,57,64–66^ whereas two studies conducted in Australia found the opposite.^67,68^ These studies found that the presence of a suicide plan was associated with greater stigma towards those who die by suicide,^67^ and those who had attended the emergency department for a suicidal crisis had higher levels of public stigma towards suicide compared with the community sample.^68^ Furthermore, individuals who had attended the emergency department for a suicide attempt had higher levels of perceived stigma than their family members.^69^ Moreover, two studies^63,70^ in two separate Chinese samples found no significant correlations of suicidal ideation with public stigma towards suicide or with stigma towards suicide attempts and those bereaved by suicide. Further, Vatan et al^71^ reported conflicting findings across samples: in Turkish students, higher suicidal ideation scores were associated with higher levels of stigma towards attempted suicide, whereas the opposite was found within the American sample of students.

##### History of suicide attempt(s)

Using the Self-Stigma of Suicide Attempt Scale^72^ in a US sample of 292 individuals, researchers found that those with a suicide attempt within the past year were more likely to apply stereotypes to themselves and experience harm as a result compared with those with more historical suicide attempts.^72^ The same results were found when comparing those with multiple suicide attempts and those with only one attempt.^72^ Three studies^73–75^ found that those with a suicide attempt history had greater stigma towards suicide attempts and suicide more generally compared to those without. Among a sample of 679 individuals in the USA, which compared suicide attempt survivors’ and their families’ experiences of attending the emergency department after an attempt, researchers found that suicide attempt survivors were more likely to feel that staff made them feel punished or stigmatised owing to their suicide attempt compared with their family members (54.5 *v.* 28.8%).^69^ This is consistent with a study from Slovenia,^45^ which found that a suicidal history was associated with anticipated suicide stigma. In a Korean sample, a history of suicide attempts significantly predicted the stigma an individual held towards others who had made a suicide attempt.^76^ Conversely, five studies found the opposite or no association at all.^32,54,57,66,77^ Two studies^54,57^ found lifetime suicide attempts to be negatively associated with public stigma regarding suicide. Among students and young adults from Bangladesh, a country where suicide is still illegal, public stigma towards those who die by suicide was found to be significantly lower in those with past suicide attempts compared with those without.^66,77^ Furthermore, among 1286 Australian adults, no association was found between suicide attempt history and public suicide-related stigma.^32^

##### Those bereaved by suicide

Four studies^78–81^ investigated the differences between suicide bereavement and other sudden bereavements using the Grief Experience Questionnaire^82^ in the UK^79,81^ and Australia.^78,80^ These studies found that individuals bereaved by suicide had higher levels of suicide-related stigma, shame, guilt, rejection and responsibility than those bereaved by other sudden natural or unnatural deaths,^78–81^ and that the differences between these groups persisted over time.^78^ Two studies^53,83^ conducted in Italy investigated different aspects of the relationship between perceived suicide-related stigma and suicide among those bereaved by suicide. Among 240 people bereaved by suicide in Italy,^53^ levels of perceived stigma towards those bereaved by suicide increased as the number of days since their loved one’s suicide increased. The other study conducted in Italy^83^ found no significant differences between individuals bereaved by suicide with and without past suicide attempts in terms of their perceived suicide-related stigma scores.

One study^84^ explored differences in public suicide-related stigma between individuals bereaved by suicide and suicide attempt survivors in the USA. This study found no significant differences among those bereaved by suicide, suicide attempt survivors and those who were members of both groups in terms of the stigmatisation of suicide. Further, among those bereaved by suicide, no difference was found with regard to stigmatisation of suicide when comparing whether the loss involved an immediate family member or not. A multiple regression analysis found that those who experienced both a loss and an attempt had lower suicide-related stigma scores than those who were solely bereaved by suicide, and that feeling very close to the deceased was also associated with less suicide-related stigma.

#### Help-seeking

No papers were included which investigated suicide-related stigma as an outcome of help-seeking behaviours.

#### Grief

##### Those bereaved by suicide

Among a sample of help-seeking individuals bereaved by suicide in Italy,^53^ levels of complicated grief were not related to levels of perceived stigma towards those bereaved by suicide.

#### Mental health

##### Experience of suicide in professional and/or personal life

Among a sample of 83 suicidologists in Slovenia, personal experiences with mental illness were associated with anticipated public suicide-related stigma.^45^ This was, however, a small sample of professionals, and so the results are not necessarily generalisable. Among a larger sample of Canadian and Australian pharmacists,^59^ individuals were more likely to agree with words describing those who die by suicide as pathetic, stupid, irresponsible and cowardly if they had not suffered from or had a close relation or friend with a mental illness.

##### History of suicidal ideation and/or attempt(s)

Among a mixed sample including people from the general population, people with a mental health condition, a suicide attempt history and those bereaved by suicide, those with a mental health condition stigmatised suicide attempts and those bereaved by suicide more than the general population.^74^ Further, among 67 individuals in Italy,^75^ higher levels of psychological distress were associated with higher levels of perceived stigma towards suicide attempts among those with a history of past suicide attempts. The study found no link between psychological distress and perceived stigma towards suicide attempts among individuals without a history of past suicide attempt. Two studies^72,85^ found that higher levels of self-stigma or perceived stigma towards suicide and suicide attempts were associated with higher levels of depression. Furthermore, self-stigma towards suicide attempts was associated with decreased recovery, self-esteem and empowerment among a US sample of 292 individuals.^72^ Only two studies^67,68^ found psychological distress to be associated with lower stigmatisation scores among those with experiences of suicidal behaviours. They were also the only studies to measure public stigma towards those who die by suicide, rather than suicide attempts and/or self-stigma of personal suicidal thoughts or attempts.

##### Those bereaved by suicide

A study comprising 155 individuals bereaved by suicide in Italy^83^ found levels of psychological distress and suffering to be positively associated with levels of perceived stigma towards those bereaved by suicide. This link persisted even after accounting for factors such as time since suicide, relationship to the decedent and demographic factors in both studies.

##### General population

Three studies conducted in Korea, China, and a mixed sample from Turkey and America^63,71,73^ found that higher levels of psychological distress and/or depression were associated with higher levels of public stigma towards suicide and perceived stigma towards suicide attempts. On the contrary, three studies conducted with the general population^32^ and among university students^54,86^ found public suicide-related stigma to be lower in those with personal experiences of mental illness.^32,54,86^ Research conducted with a sample of 290 individuals in Malaysia^86^ used the Attitude Towards Suicide^87^ scale to measure a range of different attitudes towards suicide and found that those with a family history of psychiatric illness were less likely to agree with the stigmatising statement ‘suicide should not be committed, and it is a taboo’ compared with those without such a family history.^86^ However, in a study of 224 Chinese students^70^ and another of 319 medical students from Nepal,^88^ there were no associations between depression or anxiety and public stigma towards suicide.

### What are people’s experiences of suicide-related stigma in the context of suicide risk, help-seeking, grief and mental health?

#### Suicide risk (suicide, suicidal thoughts and suicidal behaviours)

##### Experience of suicide in professional and/or personal life

Four studies^89–92^ investigated the relationship between suicide and suicide-related stigma among those with experiences of suicide. Mugisha et al^90^ investigated the relationship between public suicide-related stigma and suicide among the Baganda, a religious group based in Uganda. This study reported that the house where a person who died by suicide previously lived is often destroyed with all of its contents, as the property is seen as ‘socially infectious’. This study also found that the burial site is viewed as dangerous, with one participant stating: ‘Suicide is a danger including the burial site’. This quote also highlights the idea that suicide is a danger to those around the individual who has died by suicide. This idea of social transmission was also expressed in three other studies,^6,93,94^ which investigated the relationship among those bereaved by suicide^93,94^ and suicide attempt survivors.^6^ In addition, a sample of 73 American Indian/Native youth, adults and elders in the USA were asked about suicide and reported mixed findings, with many saying that suicide was both normalised and stigmatised.^89^ Indeed, they argued that this dual understanding meant youth felt accustomed to suicide but also ashamed to talk about it.^89^ A study conducted in Ghana,^91^ among a sample of nine clinical psychologists and eight emergency ward nurses, found that nurses viewed suicide as a sinful criminal act. They also viewed those who were suicidal as ‘wicked’ and ‘crazy’, and they blamed the suicidal person: ‘I make them feel guilty that what they are doing is a criminal offence’. By contrast, the psychologists were more likely to be empathetic and supportive, disagreeing with the view of suicide as a criminal act. Similar results were found among a sample of ten community leaders in Ghana,^92^ who reported viewing suicide as a criminal and immoral act that leads to anger among the community towards the family of those who have lost a loved one to suicide as well as the person who has attempted or died by suicide.

##### History of suicidal ideation and/or attempt

In total, 16 studies described the experiences of shame^95,96^ and labelling^6,69,93,95,97–107^ felt by individuals with a history of suicidal ideation or suicide attempt(s). For example, one suicide attempt survivor stated, ‘It was sort of like I brought shame upon my family and stuff like that’,^95^ and this shame may have led to the rejection of suicide attempt survivors by their family members, as was found in a sample of 20 individuals.^95^ The fear of being stigmatised was also commonly reported across four studies;^93,97,100,101^ for example, ‘…they assume that I’m not normal, mad or crazy’.^97^ Seven studies^6,69,95,99–101,105^ also reported that many who had attempted suicide were labelled as ‘attention seeking’, ‘stupid’ or ‘weak’ by those around them; for example, ‘they told me I just did it for attention’.^69^ Another common stereotype was that those who attempt suicide are selfish or doing it to hurt others: ‘only selfish people die by suicide’^98^ and ‘How dare you do something… we are trying so hard to help you?’.^101^ These stereotypes often appeared to be internalised, as studies^6,95,100,101,103,107^ found that participants viewed their attempt as something that signified that they were ‘selfish’, ‘crazy’, ‘silly’, ‘weak’, ‘useless’, ‘damaged’, ‘soft in the head’ or a ‘loser’.

Related to the findings above, eight studies^6,93,95–97,100,101,107^ found that among those with a suicidal history, stigmatising reactions from those around them often led to the internalisation of these suicide-related stigmas. To avoid these stigmatising attitudes, those with a suicidal history often chose to conceal their suicidal thoughts or behaviours. These studies were conducted in a range of countries with different cultural backgrounds (USA, Germany, Iran and Australia), but they all reported similar results. The following quotes highlight these findings: ‘I keep it in… I think there’s something about it that might make me feel like its expressing vulnerability or weakness’,^95^ ‘It’s embarrassing’.^96^ This secrecy and desire to conceal a suicide attempt was also described by family members of those who attempted suicide.^99^

Nine studies^6,93,98,100,101,106–109^ found that those who were suicidal experienced distancing from others or had distanced themselves from others to avoid suicide-related stigma, thereby perpetuating secrecy. One participant in a study conducted in the USA^98^ stated that her mother said: ‘If you’re going to be this way, I don’t want to know about it’. Others experienced ostracism and rejection from friends and family following an attempt^97,101,107–109^: ‘I was told I was not part of the family… even my friends did not welcome me into their fold… I felt abandoned because it was like no one wanted to associate himself with a person who wanted to kill himself’^108^ and ‘A community can be so stigmatizing… they can actually further isolate that person who has a suicidal past’.^107^ Three studies,^98,101,109^ conducted in Turkey, Australia and the USA, found that after an attempt, individuals often felt blamed and misunderstood, which may have led to further feelings of rejection and ostracism: ‘I felt even more misunderstood… they put the blame on me’.^98^

In a sample of 679 US citizens, the experiences of suicide attempt survivors and their family members when attending the emergency department after an attempt were investigated.^69^ Suicide attempt survivors reported that nurses held views that suicide was sinful and a crime: ‘Didn’t I know I was committing a sin?’. Another participant reported that hospital staff ‘…treated me like a criminal’. Family members reported hearing similar stigmatising views: ‘nurses scolded [my mother] …telling her she will end up in hell and asking how she could do that to her family’.^69^ A further five^96–98,100,110^ studies reported stigmatising beliefs that suicide was a sin or morally wrong. Two studies^93,99^ found that doctors were also judgemental of suicide attempt survivors; for example, one participant stated: ‘One doctor said, “Get him out of here. He doesn’t deserve to be here”…’.^99^ This stigmatisation of suicide was also highlighted by numerous quotes from a study conducted in Australia,^105^ which investigated the experiences of 35 individuals attending the emergency department with suicidal thoughts or behaviours; these quotes included: ‘…we can’t find a bed for you. I’m sorry you’re gonna have to go home because this isn’t a hotel’. On the contrary, a participant from another study conducted in Australia^99^ stated that a doctor tried to destigmatise an attempt to her family members: ‘She had been blaming me and the doctor said, “No stop this is her illness”’. Three studies^6,101,105^ reported the stigmatising view that those who attempt suicide are unable to recover; a family member of a suicide attempt survivor stated that a clinician told them ‘Once suicidal, always suicidal’,^101^ and, when attending the emergency department for suicidal thoughts, one participant said in relation to doctors ‘they just say “oh, you again”’.^105^

Four studies^6,93,95,101^ found that discrimination in the workplace as a result of suicide-related stigma was commonly reported among those with experiences of suicidal thoughts and/or attempts. For example, a study conducted among those with a history of ideation and/or attempts^95^ included quotes such as ‘And if you’re an employee there, clearly you don’t say that [you are suicidal] because if you do, you’re going to get fired or something like that’. Among Iranian women, suicide is viewed as unacceptable; however, stigmatising attitudes towards suicide in this group manifested slightly differently compared with other samples included in this review. For example, women who had attempted suicide were believed to have been having extra-marital affairs: ‘people will say that you have had extra marital sexual relationships!’.^97^ In Ghana, suicide is condemned, and those who attempted suicide reported being physically attacked as a result: ‘They beat me up mercilessly, hitting me with all kinds of objects…’.^108^ Similar findings were reported in a study of suicide attempt survivors in Australia; one participant stated ‘after the suicide attempt he actually treated me really badly. I was in a domestic violence situation… he was nastier than ever to me’.^101^

##### Those bereaved by suicide

Individuals bereaved by suicide also experience public suicide-related stigma, as is made explicit by quotes extracted from three studies^111,113^: ‘I certainly have felt the stigma of suicide’,^111^ ‘Somehow it is still a taboo topic’,^112^ and ‘suicide is still taboo’.^113^ Twenty-one studies^6,94,102,104,111,112,114,128^ explored the experience of those bereaved by suicide with respect to suicide-related stigma. Fourteen studies^94,102,104,112,114,116,118,121,123,127,128^ found that those bereaved by suicide reported experiencing suicide-related stigma in the form of feeling blamed, shamed, judged and isolated after their suicide loss. As a result, individuals bereaved by suicide chose to keep the suicide a secret to avoid these stigmatising attitudes.^6,94,115,116,119,120,125,126^ In a sample of ten people bereaved by suicide,^119^ this was demonstrated by quotes such as ‘suicide is like just a stigma thing, people look down on you when you tell them how he died’. Similarly, in Taiwan, suicide is seen as shameful, and a person who dies by suicide is expelled from family ancestry as punishment.^122^ Suicide-related stigma often led to social awkwardness^111,115^ and those bereaved by suicide isolating themselves and feeling alone and unsupported by those around them.^94,102,104,111,114,119,121,123,125,127^ For example, a participant in a study conducted in Australia^121^ stated: ‘I felt a little bit rejected and a bit isolated’, and in another study in the UK,^111^ participants reported being avoided by others: ‘people will walk across the other side of the street sometimes to avoid talking to you…’. Seven studies^102,104,112,119,123,127,128^ found that individuals bereaved by suicide felt blamed for the death, as in the following quote ‘…they blame me’.^102^ Contrary to this was a quote from a study conducted in Germany^104^: ‘they encouraged us and told us we are not to blame’, again indicating individual differences both between and within studies.

Similar to studies conducted with suicide attempt survivors and those experiencing suicidal ideation, five studies^6,102,104,112,118^ reported that those bereaved by suicide also experienced being labelled as ‘loony’, ‘unstable’, ‘weak’, ‘cursed’, ‘dysfunctional’ or ‘abnormal’ because of their association with suicide. Again, these labels led to secrecy – ‘You can’t talk about it – lest they will label you unstable’^102^ – or a wish that a diagnosis of suicide was not given for the death of their relation, as in a prospective study conducted in Nigeria.^118^ Parents whose children had died by suicide found it difficult to talk about because of suicide-related stigma; one participant in a study conducted in Australia^117^ stated: ‘It is one of those… an embarrassing situation, and stigma still exists… it is very hard as a parent to accept’. Five studies^102,118,123,124,127^ reported suicide as being viewed as sinful, immoral and wrong. In one study conducted in the USA,^102^ a suicide survivor discussed the stigmatising language used around suicide and how this could lead to beliefs that it is morally wrong, illegal or a sin: ‘I couldn’t stand to hear committed suicide. It felt like [my loved one] committed a crime – robbery, rape, or murder’. Further, in two other studies,^123,124^ Islamic individuals bereaved by suicide voiced the influence their religion had on their beliefs around suicide: ‘In our religion suicide is prohibited’.^123^ Like suicide attempt survivors, those bereaved by suicide reported experiencing discrimination in the workplace,^102,111,112,121^ for example: ‘I certainly have felt the stigma of suicide… I’ve felt it within my work colleagues’.^111^

People bereaved by suicide also expressed fears with regard to those who they had lost to suicide being labelled negatively.^102,104,112^ Those who had lost a loved one to suicide reported being told that the deceased was selfish; one participant stated, ‘others said that he was totally selfish’, and another stated ‘…people said that if they were me, they would hate him because what he did to me was not fair’.^104^ Further, research conducted with a sample of 30 individuals in China^126^ found that those who knew someone who died by suicide could often hold negative opinions of the individual. For example, some individuals bereaved by suicide distanced themselves from those they knew who died by suicide – as one participant stated, ‘I know that she is psychotic, and I am a normal person, so my world is definitely different from hers’ – and often, after a suicide, those around the individual who died changed their opinion of them: ‘My impression of him plummeted’.^126^

It may be worth highlighting that one of the studies conducted in Germany,^112^ with an all-female sample, found that although all participants talked about fearing negative judgement, only around a quarter had experienced judgemental reactions. This highlights the negative influence that anticipated suicide-related stigma can have on individuals affected by suicide. In three studies,^93,103,112^ some participants from a range of backgrounds (suicide attempt survivors, those bereaved by suicide) stated that talking about suicide was important to expel the stigma surrounding it, despite the negative social consequences. Participants in a study conducted in the USA^71^ stated that by disclosing their suicide attempt they were trying to challenge suicide-related stigma, which they hoped would help survivors of an attempt to manage their internalised suicide-related stigma. One participant explained: ‘The more we talk about it and the more people hear it, the less taboo it becomes, the less silent people are about it, and the less stigma’.^93^ Further, a study conducted in Canada^103^ highlighted the importance of talking about suicide within a peer support group, to overcome internalised suicide-related stigma: ‘I do have some value to bring from my experiences’. Similar results were found among those bereaved by suicide; one participant stated regarding their reason for sharing their loss: ‘I want people to realize that suicide is quite common, so that it is no longer taboo to talk about it’.^112^

#### Help-seeking

##### Experience of suicide in professional and/or personal life

Research conducted with 73 individuals from the American Indian and Alaskan Native populations^89^ investigated the association between help-seeking and suicide-related stigma. One of the key findings from this study was that youth do not report feelings of suicidality because they are ‘scared to talk about it’ owing to stigmatising feelings of disgust and stupidity.

##### History of suicidal ideation and/or attempt(s)

Thirteen studies^99,101,103,105,106,109,113,114,125,129,131^ focused on those with personal experiences of suicidal thoughts and/or behaviours. These studies reported that the reasons these individuals had for not disclosing their suicidal thoughts or behaviours to both formal and informal sources of help centred around avoiding suicide-related stigma and staying silent. This silence was due to their experiences with feelings of shame, embarrassment, judgement and rejection, as well as a general fear surrounding talking about suicide. These findings are highlighted by quotes such as: ‘I was ashamed… So I decided not to tell anyone’,^100^ and, when one participant was asked why they did not seek help, they simply stated: ‘shame’.^130^ Further, two studies^130,131^ found that participants did not disclose suicidal ideation or intent because they were worried about being labelled as ‘crazy’; participants stated: ‘they might think I was crazy or something’.^130^ The prospect of being labelled crazy was linked to a fear of hospital admission and severe treatment, as described by participants in three studies^100,125,132^: ‘I was scared they will put me into a locked ward’.^100^ There was also a shame associated with attempting suicide, as highlighted by the following quote: ‘I don’t tell anybody because you feel stupid, because you didn’t see through what you intended’.^99^ These results indicate that those in need of help use silencing and avoidant behaviours to avoid feeling stigmatised.

Help-seeking experiences are not always positive, owing to the stigmatisation of suicide by healthcare professionals. These attitudes can often be internalised, as was found by three studies^100,103,105^ involving suicide attempt survivors in three different countries (Germany, Canada and Australia). This internalisation could lead to the individual wanting to cope with suicidality on their own; indeed, one participant in a study in Germany^100^ stated: ‘I realized I have to deal with it myself. I decided not to seek professional help or talk to others about it anymore’. Similarly, in studies conducted in Canada^103^ and the UK,^106^ negative reactions were experienced within a hospital setting; one participant shared that her ‘negative experiences with the system were confirmation that I’m worthless’, and, upon hearing similar experiences within the focus groups, she thought ‘maybe I deserve better too’.^103^ A study conducted in Australia^105^ further emphasised the dangers of negative help-seeking experiences, as young people experiencing suicidal thoughts or behaviours reported that if they had negative experiences at the emergency department they would be deterred from attending again in the future, increasing their suicide risk. Among the German sample,^100^ two participants did not discuss avoidant behaviour in response to suicide-related stigma and reported less distress; these participants may not have internalised the stigma associated with suicide like the others quoted above.

##### Those bereaved by suicide

Among people bereaved by suicide, two studies^114,118^ found that shame related to the loss of a loved one to suicide prohibited participants from seeking help for their own health concerns. For example, in a study conducted in Ireland,^114^ participants bereaved by suicide did not want to sit in a general practitioner’s waiting room as a result of feeling ashamed about their loss to suicide, and in one instance backdoor entry was arranged ‘so that no one could see her’.^114^ This pattern was also found among a Nigerian sample,^118^ as is highlighted by the following quote: ‘If any of us now will even go to the hospital for anything, people will say that another of their brothers wanted to kill himself’.^118^ As a result of the stigmatisation of suicide, participants found it hard to accept the death and believed accepting help would be accepting the death: ‘Help… for what?… It means that I agree that my relation hanged himself’.^118^

##### General population

Among male prisoners in New Zealand, negative labelling of those reporting suicidal thoughts, such as ‘attention-seeking’ and ‘mentally weak’, contributed to the men not seeking help for their suicidal thoughts.^132^

#### Grief

##### Those bereaved by suicide

Four studies^90,104,122,124^ found that those who had lost a loved one to suicide often chose to keep the death a secret and struggled to grieve openly owing to the stigmatisation of suicide. In a study conducted among the Taiwanese community,^122^ one participant stated: ‘We kept a low profile to hold that funeral… my mother did not die naturally, my father wanted to keep it low profile…’, highlighting the secrecy surrounding suicide. Further, research conducted among the Baganda population (a religious group in Uganda) uncovered an unwritten law within the community that meant they were expected to grieve inwardly if they lost a loved one to suicide, as described in the following quote: ‘When one commits suicide, within our tradition, you are not allowed to shed tears as a sign that you are not in approval of the act’.^90^ Moreover, after a death, the Baganda use songs and sayings which usually devalue the person who died by suicide, and the usual grieving period is not allowed; instead, the deceased must be buried immediately.^90^ Similarly, in two other studies,^104,124^ the same point was made; one individual bereaved by suicide recalled being told: ‘This is a suicide, don’t be sad’.

##### Mental health

No qualitative studies that investigated people’s experiences of suicide-related stigma and mental health were included in this review.

### Key themes

Table 2 highlights the key themes identified from the qualitative literature across groups. Furthermore, we felt it would be useful to map the relevant quantitative findings on to this table to emphasise that quantitative and qualitative studies reported similar results. Qualitative themes are displayed with a circle, and quantitative findings are displayed with a square; where both are present, this indicates a common finding across the qualitative and quantitative studies.

## Discussion

This is the first scoping review to synthesise and compare the findings of studies across several groups affected by suicidal thoughts and behaviours. We reported the findings of 100 studies that investigated suicide-related stigma and suicidal thoughts and/or behaviours, mental health, help-seeking and/or grief. The review included studies in which suicide-related stigma was the exposure variable and those in which suicide-related stigma was the outcome variable. We have developed a model explaining the associations between suicide-related stigma and help-seeking, grief, suicide and mental health based on the findings of this review (Fig. 2). This model succinctly explains the results relating to the three research questions of this review, linking the qualitative findings with the quantitative findings. The first research question was answered using findings from studies which investigated suicide-related stigma as the exposure variable. The second research question was addressed with studies which investigated suicide-related stigma as the outcome variable, and the third research question was answered using the findings of qualitative studies. This review highlighted the potential risks of suicide-related stigma in relation to suicidal thoughts and/or behaviours, mental health, help-seeking and grief. However, it also identified some evidence of suicide-related stigma being protective. Notably, the year of publication does not seem to be an explanation for the differences in findings, as papers published more than 15 years apart reported similar results i.e. a study from 2002^79^ had similar results to those from 2019 and 2020.^78,80^

### Suicidal thoughts and behaviours

In studies in which suicide-related stigma was the exposure variable, among those with a history of suicide attempts, those bereaved by suicide and the general population, both public and perceived suicide-related stigma was generally associated with a higher risk of suicidal thoughts and behaviour, as well as with feelings of guilt, shame and secrecy attached to suicide attempt(s). Only one study found public suicide-related stigma to decrease the chances of self-harm and suicidal behaviour; however, the reasons for this are unclear, as this sample was similar to those of studies which found the opposite. Where suicide-related stigma was considered the outcome variable, personal experiences of past suicide attempts were generally associated with higher levels of stigma towards suicide, suicide attempts, and anticipated stigma about one’s own suicidality, which could arguably be a risk factor for future suicide attempts. Qualitative studies highlighted the reasons that suicide-related stigma may lead to a heightened risk of suicide: suicide-related stigma reportedly caused feelings of shame, blame, guilt and labelling, which led to a fear of opening up about suicide and reduced help-seeking behaviours. These negative consequences of suicide-related stigma may also lead to social withdrawal and isolation; this is in line with the propositions of the MLT,^10^ such as labelling being associated with discrimination and exclusion of individuals from society. These negative consequences could also lead to an increased risk of suicide and self-harm, given that feelings of isolation and shame are associated with feelings of defeat and entrapment, which are precursors to suicidal ideation as explained by the IMV model.^22^

Experience of suicide in an individual’s personal life and/or a history of suicidal ideation was most commonly associated with less stigma towards those who die by suicide. It seems that those with a history of suicide attempt(s) and those bereaved by suicide perceived higher levels of suicide-related stigma, and they also held more stigmatising views towards suicide compared with those with experiences of suicide and/or a history of suicidal ideation. Findings did differ across studies, and some found there to be no association between suicide and suicide-related stigma. The reasons for these differences are unclear owing to the high levels of heterogeneity across the studies. A possible explanation among those with a history of suicide attempts is that none of the studies which found suicide-related stigma to be higher among suicide attempt survivors used the SOSS-SF to measure suicide-related stigma, whereas every study that found no association or lower suicide-related stigma in suicide attempt survivors used the SOSS-SF. This suggests that the discrepancy may be related to some aspect of the SOSS-SF scale; however, this scale has been shown to have high internal consistency, good validity, good factor convergence^54^ and robust psychometric properties^32,67^ across a range of samples. Cultural differences may have contributed to the differences among findings. For instance, the study by Vatan et al^71^ used a sample of both Turkish and American students, and findings differed between these two groups. It is also worth noting that both studies which found no association between suicidal ideation and suicide-related stigma were conducted with Chinese participants.

### Mental health

All of the studies which investigated suicide-related stigma as the exposure variable found that all three types of stigma (public, self and perceived) were associated with higher levels of depression and psychological distress. However, studies which investigated suicide-related stigma as an outcome of mental health differed in their findings. Among those with experience of suicide or those bereaved by suicide, higher levels of psychological distress led to higher levels of public and perceived suicide-related stigma. Where suicide-related stigma was the outcome variable, findings were mixed among those with a history of suicidal ideation and/or attempts and the general population. There does not seem to be a clear reason for the differing findings across the 22 studies. However, these studies were conducted in a range of different countries and samples, using a range of different methods. Most studies (all except one) which found levels of psychological distress or mental health concerns to be associated with lower suicide-related stigma or no association at all used the SOSS-SF,^86^ whereas none of the studies which found psychological distress or mental health concerns to be associated with higher suicide-related stigma or vice versa used the SOSS-SF to measure suicide-related stigma. Therefore, it could be argued that the differences are linked to whether a study used the SOSS to measure suicide-related stigma, as the type of suicide-related stigma measured could have affected the findings. For instance, studies investigating perceived and/or experienced stigma towards suicide attempts or those bereaved by suicide (i.e. studies using measures other than the SOSS) found higher levels of psychological distress to be associated with higher levels of suicide-related stigma or vice versa.

### Help-seeking

In all the quantitative studies included in this review, suicide-related stigma was the exposure variable, and most of these studies (*n* = 6) highlighted the predominantly negative effects of suicide-related stigma on help-seeking intentions and behaviours. Two studies found that suicide-related stigma encouraged help-seeking; notably, one study found suicide-related stigma to encourage help-seeking from professional sources but decrease help-seeking from friends and family.^43^ The qualitative literature enabled a deeper understanding of why suicide-related stigma was generally found to lower help-seeking intentions. Participants in these studies reported feelings of shame, embarrassment and fear surrounding other people’s reactions to suicide as a result of suicide-related stigma and described how these feelings silenced both those experiencing suicidal thoughts or behaviours and those bereaved by suicide and deterred them from seeking help. Furthermore, the negative labels that are often associated with suicide and those who experience suicidal thoughts or attempt(s) – for example, that they are ‘attention-seeking’ or ‘weak’ – are also worth considering as reasons individuals might not seek help. Reasons for the differences in findings across the ten quantitative papers investigating help-seeking could be related to demographic differences within the cohorts, such as education level or age, as well as cultural differences among countries (Jordan and Hong Kong versus Jakarta); the papers also differed with respect to how they measured both help-seeking and suicide-related stigma.

### Grief

With regard to those bereaved by suicide, this review found similar results to the previous reviews mentioned earlier, by Hanschmidt et al^9^ and Evans & Abrahamson.^28^ Two studies in our review^38,52^ found that perceived stigma towards those bereaved by suicide was associated with greater grief-related difficulties and secrecy. This was further supported by the qualitative studies, which found that individuals bereaved by suicide experienced stigmatisation from numerous groups, and that this stigmatisation led to negative consequences such as secrecy, social withdrawal and reduced help-seeking behaviours. People bereaved by suicide were found to experience grieving difficulties as a result of stigmatisation, such as being told that suicide was a sin and, in some cultures, not being allowed to grieve outwardly, as in the Baganda community. People bereaved by suicide reported feeling rejected and socially isolated following the suicide, and studies found that the stigmatisation of suicide also resulted in poorer mental health among this population. The only study to consider suicide-related stigma as the outcome variable was also the only study to find no association^53^ between help-seeking and suicide-related stigma. This study focused on help-seeking people bereaved by suicide rather than everyone bereaved by suicide; there may have been lower levels of suicide-related stigma in this sample, as it has been shown that suicide-related stigma reduces help-seeking behaviours. All three of the quantitative papers investigating the relationship between suicide-related stigma and grief had samples largely comprising female participants, and so these results are not necessarily generalisable to a population of males bereaved by suicide.

### Strengths and limitations

This review was conducted using a robust search strategy and following the PRISMA 2018 guidelines.^29^ Four reviewers (J.M.W., D.S., M.E.E. and N.B.) were involved at the screening and quality assessment stages to ensure any bias was reduced and articles were thoroughly assessed. This review was inclusive of both qualitative and quantitative study designs, enabling deeper understanding and investigation into the association between suicide-related stigma and the four variables explored within this review. Furthermore, this review is the first of its kind, as previous reviews in this area have focused on those bereaved by suicide; therefore, it provides a clearer picture of other groups affected by suicide and their experiences of suicide-related stigma. This review also synthesises the relationship between suicide-related stigma and a number of factors such as help-seeking and mental health, again advancing our understanding of the effects of suicide-related stigma across a range of groups.

Studies included within this review had a range of shortcomings and limitations. The voluntary nature of all the included studies meant that those participating may have had an interest in suicide research, leading to potential bias. Further, the results may not be generalisable to those who have experienced suicidal thoughts and/or behaviours or the loss of a loved one to suicide and have not spoken out about their experience or sought help owing to factors such as suicide-related stigma. Most of the studies included within this review were cross-sectional; therefore, it was not possible to determine causality.

### Implications

It is clear from this review that the stigma associated with suicide is felt and experienced by several groups affected by suicide, often negatively and sometimes with tragic consequences. It is also clear that suicide-related stigma can be exacerbated by experiences of suicide, by people around the person affected by suicide and, more worryingly, by healthcare professionals. Some of the contradictory findings highlight a need for more research in this area to understand why different cultures and populations differ regarding their experiences of suicide-related stigma. Moreover, conducting future research in this area globally would be conducive to gaining a deeper understanding of the cultural differences, as most of the existing research has been conducted in the global north. It would also be worth conducting more research within general population samples to establish why individuals stigmatise suicide and how this affects their attitudes towards those who have suicidal experiences or how their levels of suicide-related stigma may influence their potential for future suicidal behaviour longitudinally.

It is also worth noting that the findings in this review support the arguments for both of the previously mentioned models of mental health stigma (the MLT^10^ and the social cognitive model^11,12^), as they both highlight how stigma towards mental illness is related to labelling and/or stereotyping, exclusion and discrimination. To build on this, we propose a model of suicide-related stigma, informed by the findings of this review, to explain why suicide-related stigma may lead to poor mental health, lowered help-seeking, grief difficulties and increased suicide risk. It is hoped that this model will inform future intervention and prevention efforts.

This scoping review also highlights the importance of allowing those affected by suicide to openly discuss their experiences, as several participants indicated that talking about suicide was important in reducing the ‘taboo’ around the topic; it also enabled positive reflections among these individuals. With this in mind, it would be useful for future researchers in this area to work closely with those affected by suicide, not only to ensure that research methods are sensitive and appropriate but also to empower these individuals and break down barriers and stigmas related to suicide.

Despite some differences among study findings, most of the studies included in this review reported predominantly negative consequences of suicide-related stigma worldwide, irrespective of participants’ background. Furthermore, these consequences were associated with heightened risks of suicide, isolation, poor mental health, lowered help-seeking behaviours and complicated grief. Therefore, tackling the stigma associated with suicide may improve outcomes for individuals affected by suicidal thoughts and behaviours. Concealment of suicidal loss and suicidal behaviours because of suicide-related stigma seems to exacerbate these negative consequences, and, as noted by a few participants, talking about the stigma of suicide helps to reduce the taboo around the subject. The high levels of heterogeneity across the studies should be taken into consideration when drawing conclusions. Indeed, much more research is needed to ensure that we have a clear picture of the implications as well as the causes of suicide-related stigma.

## Supporting information

Wyllie et al. supplementary material 1 Wyllie et al. supplementary material

Wyllie et al. supplementary material 2 Wyllie et al. supplementary material

Wyllie et al. supplementary material 3 Wyllie et al. supplementary material

## Data Availability

Data availability is not applicable to this article as no new data were created or analysed in this study.

## Supplementary material

The supplementary material is available online at https://doi.org/10.1192/bjo.2024.857.
