# Supplementary material for: Suicide-related stigma and its relationship with help-seeking, mental health, suicidality and grief: scoping review
Source: BJPsych Open. 2025 Mar 21;11(2):e60. doi: 10.1192/bjo.2024.857 (PMC12001961; doi:10.1192/bjo.2024.857)
Supplement: Wyllie et al. supplementary material 2 — Wyllie et al. supplementary material [file S2056472424008573sup002.docx]

| **#**  **Appendix 2. Search Strategy for APA PsycInfo** | **Query** | **Limiters/Expanders** |
| --- | --- | --- |
| S11 | S5 AND S10 | Expanders - Apply equivalent subjects Search modes - Boolean/Phrase |
| S10 | S6 OR S7 OR S8 OR S9 | Expanders - Apply equivalent subjects Search modes - Boolean/Phrase |
| S9 | TI attitud* OR AB attitud* | Expanders - Apply equivalent subjects Search modes - Boolean/Phrase |
| S8 | DE "Attitudes" OR DE "Prejudice" | Expanders - Apply equivalent subjects Search modes - Boolean/Phrase |
| S7 | TI stigma* OR AB stigma* | Expanders - Apply equivalent subjects Search modes - Boolean/Phrase |
| S6 | DE "Stigma" OR DE "Mental Health Stigma" OR DE "Self-Stigma" OR DE "Attitudes" OR DE "Explicit Attitudes" OR DE "Implicit Attitudes" OR DE "Social Discrimination" OR DE "Stereotyped Attitudes" | Expanders - Apply equivalent subjects Search modes - Boolean/Phrase |
| S5 | S1 OR S2 OR S3 OR S4 | Expanders - Apply equivalent subjects Search modes - Boolean/Phrase |
| S4 | TI suicid* OR AB suicid* | Expanders - Apply equivalent subjects Search modes - Boolean/Phrase |
| S3 | DE "Self-Poisoning" | Expanders - Apply equivalent subjects Search modes - Boolean/Phrase |
| S2 | DE "Suicidal Ideation" OR DE "Suicidal Behavior" OR DE "Attempted Suicide" OR DE "Suicidality" | Expanders - Apply equivalent subjects Search modes - Boolean/Phrase |
| S1 | DE "Suicide" OR DE "Self-Injurious Behavior" OR DE "Suicidality" OR DE "Suicide Prevention" OR DE "Suicidology" | Expanders - Apply equivalent subjects Search modes - Boolean/Phrase |

*Appendix 2. The search strategy used for the APA PsycInfo database*
