# Supplementary material for: Suicide-related stigma and its relationship with help-seeking, mental health, suicidality and grief: scoping review
Source: BJPsych Open. 2025 Mar 21;11(2):e60. doi: 10.1192/bjo.2024.857 (PMC12001961; doi:10.1192/bjo.2024.857)
Supplement: Wyllie et al. supplementary material 3 — Wyllie et al. supplementary material [file S2056472424008573sup003.docx]

**Data Extraction Sheet**

Title:

Authors:

Publication Date:

Type of Study:

| **Demographic Information** | |
| --- | --- |
| *Age* |  |
| *Gender* |  |
| *Ethnicity* |  |
| *Marital Status* |  |
| *Religion* |  |
| *Where was study done?* |  |
| *Population* |  |
| *Sample Size* |  |

| **Measures** | |
| --- | --- |
| *Measures used* |  |
| *Reliability* |  |
| *Type of stigma measured* |  |

| **Outcomes** | |
| --- | --- |
| Impact of stigma on help-seeking |  |
| Impact of stigma on grieving |  |
| Impact of stigma on mental health/vice versa |  |
| Impact of stigma on suicide/vice versa |  |
| *Other possible interesting information* |  |

| **Other information** | |
| --- | --- |
| *Authors Limitations* |  |
| *Sub-group Analyses* |  |
